# Rhesus Monkeys (*Macaca mulatta*) Detect Rhythmic Groups in Music, but Not the Beat

**DOI:** 10.1371/journal.pone.0051369

**Published:** 2012-12-12

**Authors:** Henkjan Honing, Hugo Merchant, Gábor P. Háden, Luis Prado, Ramón Bartolo

**Affiliations:** 1 Cognitive Science Center Amsterdam, Institute for Logic, Language and Computation, University of Amsterdam, Amsterdam, The Netherlands; 2 Department of Cognitive Neuroscience, Instituto de Neurobiología, Universidad Nacional Autonoma de México, Campus Juriquila, Querétaro, México; Northwestern University, United States of America

## Abstract

It was recently shown that rhythmic entrainment, long considered a human-specific mechanism, can be demonstrated in a selected group of bird species, and, somewhat surprisingly, not in more closely related species such as nonhuman primates. This observation supports the *vocal learning hypothesis* that suggests rhythmic entrainment to be a by-product of the vocal learning mechanisms that are shared by several bird and mammal species, including humans, but that are only weakly developed, or missing entirely, in nonhuman primates. To test this hypothesis we measured auditory event-related potentials (ERPs) in two rhesus monkeys (*Macaca mulatta*), probing a well-documented component in humans, the mismatch negativity (MMN) to study rhythmic expectation. We demonstrate for the first time in rhesus monkeys that, in response to infrequent deviants in pitch that were presented in a continuous sound stream using an oddball paradigm, a comparable ERP component can be detected with negative deflections in early latencies (Experiment 1). Subsequently we tested whether rhesus monkeys can detect gaps (omissions at random positions in the sound stream; Experiment 2) and, using more complex stimuli, also the beat (omissions at the first position of a musical unit, i.e. the ‘downbeat’; Experiment 3). In contrast to what has been shown in human adults and newborns (using identical stimuli and experimental paradigm), the results suggest that rhesus monkeys are not able to detect the beat in music. These findings are in support of the hypothesis that *beat induction* (the cognitive mechanism that supports the perception of a regular pulse from a varying rhythm) is species-specific and absent in nonhuman primates. In addition, the findings support the auditory timing *dissociation hypothesis*, with rhesus monkeys being sensitive to rhythmic grouping (detecting the start of a rhythmic group), but not to the induced beat (detecting a regularity from a varying rhythm).

## Introduction

The ability to perceive a regular beat in music and synchronize to it (e.g., by foot tapping or dancing) is a common and widespread human skill [1]. It is also a skill that has been suggested to be domain-specific [2] and, arguably, conditional to the origins of music [3]. Nevertheless, it is still unclear whether this ability should be considered species-specific [4]. It was recently shown that rhythmic entrainment, long considered a human-specific mechanism, can be demonstrated in a select group of bird species [5], [6], and, somewhat surprisingly, not in more closely related species such as nonhuman primates [7]. This observation supports the *vocal learning hypothesis* [8] that suggests that rhythmic entrainment is a by-product of the vocal learning mechanisms that are shared by several bird and mammal species, including humans, but that are only weakly developed, or missing entirely, in nonhuman primates [4]. However, since no evidence of rhythmic entrainment was found in many vocal learners (including dolphins, seals, and songbirds [9]), vocal learning may be necessary, but not sufficient [4] for *beat induction* – the cognitive mechanism that supports the perception of a regular pulse from a varying rhythm [3].

In addition, there might be a dissociation between rhythm perception and beat induction, as was shown in a lesion study with humans [10]. This study suggests different cognitive mechanisms to be active for duration-based timing versus beat-based timing, with beat induction being dependent on distinct parts of the timing network in the brain [11], [12]. We hypothesize that humans share rhythm perception (or duration-based timing) with other primates, while the beat induction (or beat-based timing) is only present in specific species (including humans and a selected group of bird species [6]), arguably as a result of convergent evolution [13]. We will refer to this as the auditory timing *dissociation hypothesis.*

Most existing animal studies on rhythmic entrainment have used behavioral methods to probe the presence of beat perception, such as tapping tasks [7] or measuring head bobs [5]. However, if the production of synchronized movement to sound or music is not observed in certain species (such as in nonhuman primates, seals or dolphins [9]), this is no evidence for the absence of beat perception. It could well be that while certain species are not able to synchronize movements to a rhythm, they do have *beat induction* and as such, can perceive a beat. With behavioral methods that rely on overt motoric responses it is difficult to separate between the contribution of perception and action; more direct, electrophysiological measures such as event-related brain potentials, allow testing for neural correlates of beat perception.

In the current study, we measure auditory event-related brain potentials (ERP) in two rhesus monkeys (*Macaca mulatta*) using the mismatch negativity component (MMN) as an index of (the violation of) rhythmic expectation using an oddball paradigm [3], [14].

MMN has been investigated mainly in mice, rats and rodents (which are primarily negative), and in carnivores (cat) and primates (macaque), which have reported positive results. Most studies, however, use intracranial and single-cell recording techniques and measure stimulus-specific adaptation (SSA), an index that is similar but not identical to MMN (see [15] for a discussion). Just a few studies measured non-invasive scalp-recorded auditory event-related potentials (ERPs) in nonhuman primates, with Ueno et al. [16] being the first study, to our knowledge, to show it is possible, in principle, to measure an MMN-like response in an awake, non-sedated chimpanzee (*Pan troglodytes*).

In the current study, using oddball paradigms [3], [14], we record auditory ERPs from two rhesus monkeys (*Macaca mulatta*) utilizing the MMN as an index of the violation of (rhythmic) expectation. First we tested whether an MMN can be elicited in rhesus monkeys (using deviant tones at random positions in the sound stream; Experiment 1). Second, we investigated whether an MMN can be elicited by infrequent omissions of regular tones (inserting gaps at random positions in the sound stream; Experiment 2). Subsequently, we probed the presence of beat induction by selectively omitting parts of a musical rhythm (randomly inserting gaps at the first position of a musical unit, i.e. the ‘downbeat’; Experiment 3).

**Figure 1:**
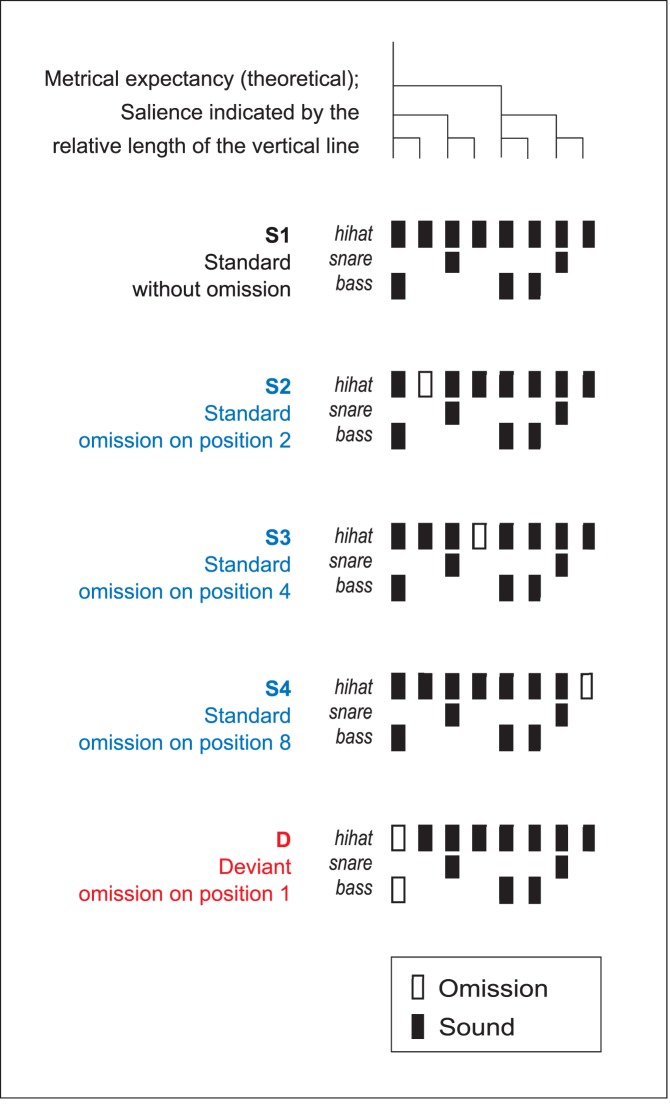
Schematic diagram of the rhythmic stimulus patterns used in Experiment 3 (Adapted from Honing *et al*., 2009).

The latter paradigm has been used previously to show sensitivity to the beat in human adults and newborns [17], [18], [19], [20]. In these studies sound sequences were used that are based on a typical 2-measure rock drum accompaniment pattern composed of snare, bass and hi-hat spanning 8 equally spaced (isochronous) positions (see Figure 1). Because the MMN is known to be elicited by deviations from temporal expectations [3], it is especially appropriate for testing beat induction. One of the most salient perceptual effects of beat induction is a strong expectation of an event at the first position of a musical unit, i.e., the ‘downbeat’. Therefore, occasionally omitting the downbeat in a sound sequence composed predominantly of strictly metrical (regular or ‘nonsyncopated’) variants of the same rhythm should elicit discriminative ERP responses, that is, if the subject extracted the beat of the sequence.

## Methods

### Ethics Statement

All the animal care, housing, experimental procedures were approved by the National University of Mexico Institutional Animal Care and Use Committee and conformed to the principles outlined in the Guide for Care and Use of Laboratory Animals (NIH, publication number 85-23, revised 1985). Both monkeys were monitored daily by the researchers and the animal care staff, and every second day from the veterinarian, to check the conditions of health and welfare. To ameliorate their condition of life we routinely introduced in the home cage (1.3 m^3^) environment toys (often containing items of food that they liked) to promote their exploratory behavior. The researcher that tested the animals spent half an hour interacting with the monkeys directly, giving for example new objects to manipulate. We think that this interaction with humans, in addition to the interaction that was part of the task performed, can help to reduce potential stress related to the experiment. Food and water where given ad libitum.

### Participants

Two rhesus monkeys participated in the ERP measurements. Aji, a 2 year old male (referred to as monkey A) and Yko, a 5 year old male (referred to as monkey Y). Both monkeys have normal hearing. They were awake (i.e. not sedated) during the measurements, sitting in a quiet room [3 (l)×2 (d)×2.5 (h) m] with dimmed lighting and two loudspeakers in front of them. The ERP measurements were performed after a morning session of unrelated behavioral experiments. The animals were seated comfortably in a monkey chair where they could freely move their hands and feet. No head fixation was used and the EEG electrodes were attached to the monkey’s scalp using tape. To ease the fixation of the electrodes, the monkey’s hair on the scalp and reference ear was shaved.

### Stimuli

In Experiment 1 pure sine-wave tones were used for the two-stimulus oddball paradigm. Their frequencies were 500 Hz and 1500 Hz, with a duration of 50 ms, and a rise and fall of 5 ms. The frequencies of these tones were within the audible range of both monkeys.

In Experiment 2 a sine-wave with a frequency 1000 Hz was used, with a duration of 50 ms and a rise and fall of 5 ms.

In Experiment 3 sound sequences based on a typical 2-measure rock drum accompaniment pattern (S_1_) were used, composed of snare, bass and hi-hat, spanning equally spaced positions (see Figure 1). Four further variants of the S_1_ pattern (S_2_–S_4_ and D) were created by omitting sounds in different positions. Within the patterns the onset-to-onset interval between successive sounds was 150 ms with 75 ms onset-to-offset interval (75 ms sound duration). Patterns in the sequence were delivered as a continuous sound stream. Loudness of the sounds was normalized so that all stimuli had the same loudness.

Sound stimuli were presented through 2 loudspeakers placed 1.1 meters away from the subject (and 1 meter apart from each other). The sound intensity measured at the subject position was approximately 60 dB SPL.

### Procedures/Experimental Design

In all three experiments an auditory oddball paradigm was used. The experimental paradigm was adapted from previous studies (Experiment 1: [16]; Experiment 2: [21]; Experiment 3: [20]).

In Experiment 1 and 2 sound inter-onset-intervals were 600 ms and 150 ms, respectively. In both experiments standards (0.9 probability) were randomly replaced (0.1 probability) with deviants and deviant omissions (i.e. silence), respectively. In Experiment 1 for half of the blocks one frequency was used as deviant and the other as standard (i.e. S_500_, D_1500_), switching roles for the other half of the blocks (i.e. S_1500_, D_500_). In Experiment 2 the inter-onset-interval was 150 ms (an interval motivated by human studies [21], and that is within the ‘preferred tempo’ range of rhesus monkeys [22]).

In Experiment 3 the 4 strictly metrical sound patterns (S_1_–S_4_; standards) made up the majority of the patterns in the sequences (0.225 probability, respectively). In the standard patterns regular omissions occurred in metrically weak positions, leaving these patterns metrically intact. Occasionally, the D pattern was delivered (0.1 probability) in which the downbeat was omitted, which interrupted the metricality of the pattern. The order of the five patterns was pseudo-randomized, enforcing at least three standard patterns between successive D patterns and no D after S_4_ to avoid two consecutive omissions. A control sequence (deviant-control) repeating the D pattern 100% of the time was also delivered (see [20] for more details).

The ERP measurements were conducted in a repeated session, containing all three experiments in random order. The monkeys participated in one recording session per day, to a total of 11 sessions for monkey A and 23 sessions for monkey Y (monkey Y moved considerably more than Monkey A). All measurement was completed in about one month per monkey. Each experiment consisted of 10 blocks with 306 repetitions for each block.

### EEG Recording and Analysis

**Figure 2:**
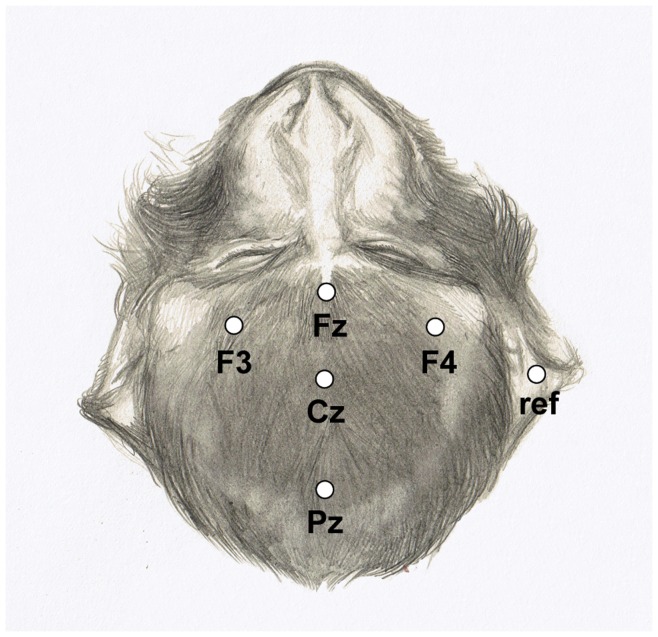
Electrode positions marked on the head of a rhesus monkey (Drawing courtesy of Roos Holleman).

The EEG was recorded from electrodes (Grass EEG electrodes; #FS-E5GH-60) attached to five scalp positions (Fz, Cz, Pz, F3, F4) according to the 10–20 system (see Figure 2).

The electrodes were connected to a Tucker-Davis Technologies (TDT) headstage (#RA16LI) for low impedance electrodes. This headstage was connected to a TDT RA16PA preamplifier, which in turn was connected to a TDT RZ2 processor. RZ2 was programmed to acquire the EEG signals with a sampling rate of 498.25 Hz and the bandpass filters were set at 0.01–100 Hz.

All electrodes were attached using Ten20 Conductive EEG Paste and medical tape, and were referenced to the right ear (fleshy part of the pinna). In the offline analysis, a 0.1–30 Hz band-pass FIR filter (Kaiser-window) was applied. With zero latency set to the onset of the stimuli, epochs of −100–500 ms (Experiment 1), 0–450 ms (Experiment 2), and 0–600 ms (Experiment 3) were extracted. All epochs were baseline corrected to zero using a 100 ms pre-stimulus interval in Experiment 1 and the whole epoch in Experiments 2 and 3. Epochs that exceeded +/−150 µV amplitude were excluded from the statistical analysis. EMG recordings were obtained from the *temporalis* muscles. No event-locked activity was found in these recordings. The number of epochs accepted for analysis for the three experiments are given in the Tables 1–4.

**Table 1:** Mean amplitudes of standard- and deviant-waves for each condition and scalp position (Experiment 1).

Monkey A				
	S_1500_(n = 21834)	D_500_(n = 2219)	S_500_(n = 20993)	D_1500_(n = 2353)
Fz	−2.0 (0.2)	−8.2 (0.5)	−2.6 (0.2)	−4.7 (0.5)
Cz	−1.2 (0.1)	−5.1 (0.5)	−1.7 (0.1)	−3.4 (0.4)
Pz	−0.3 (0.1)	−0.6 (0.5)	−0.8 (0.1)	−0.7 (0.4)
F3	−1.7 (0.1)	−6.4 (0.5)	−2.3 (0.1)	−3.7 (0.4)
F4	−1.4 (0.1)	−6.0 (0.5)	−1.9 (0.2)	−4.0 (0.4)
**Monkey Y**				
	**S_1500_** **(n = 35613)**	**D_500_** **(n = 3860)**	**S_500_** **(n = 35378)**	**D_1500_** **(n = 3797)**
Fz	−3.0 (0.1)	−7.6 (0.3)	−3.5 (0.1)	−7.0 (0.3)
Cz	−3.7 (0.1)	−7.3 (0.4)	−3.7 (0.1)	−8.9 (0.5)
Pz	−1.0 (0.2)	−1.0 (0.5)	−1.1 (0.2)	−1.4 (0.5)
F3	−2.2 (0.1)	−4.7 (0.4)	−2.2 (0.1)	−5.0 (0.4)
F4	−1.8 (0.1)	−5.1 (0.4)	−2.1 (0.1)	−3.4 (0.5)

*Note*. Mean amplitudes (µV) are indicated with *SE* values in parentheses. S: values for standard stimuli; D: values for deviant stimuli; Subscript indicates tone frequency in Hz; number of epochs (n) are indicated in parentheses. The time windows adopted are 59–109 ms for monkey A and 61–111 ms for monkey Y (See Methods).

**Table 2:** Mean amplitudes of standard- and deviant-waves for each scalp position (Experiment 2).

Monkey A				
		S (n = 13187)		D (n = 2523)
Fz		3.2 (0.2)		1.5 (0.3)
Cz		2.5 (0.2)		1.1 (0.4)
Pz		0.6 (0.2)		−0.2 (0.3)
F3		2.5 (0.1)		0.8 (0.3)
F4		2.6 (0.1)		0.7(0.3)
**Monkey Y**				
	**S (n = 22907)**		**D (n = 4402)**	
Fz	−0.8 (0.1)		−0.1 (0.2)	
Cz	−1.1 (0.1)		−0.0 (0.3)	
Pz	−1.3 (0.2)		−0.8 (0.3)	
F3	−0.5 (0.1)		−0.2 (0.3)	
F4	−0.2 (0.1)		0.2 (0.3)	

*Note*. Mean amplitudes (µV) are indicated with *SE* values in parentheses. S: values for standard stimuli; D: values for deviant stimuli (omissions), n: number of epochs. The time windows adopted are 124–174 ms for monkey A and 77–127 ms for monkey Y (See Methods).

**Table 3:** Mean amplitudes of standard- (S_1–4_), deviant- (D), and ‘deviant-control’-waves (D_control_) in the early window (just after the omission) for each stimulus type and scalp position (Experiment 3).

Monkey A						
	S_1_ (n = 5458)	S_2_ (n = 5518)	S_3_ (n = 5515)	S_4_ (n = 5421)	D (n = 2404)	D_control_ (n = 12959)
Fz	−0.2 (0.2)	0.2 (0.2)	0.2 (0.2)	0.2 (0.2)	0.3 (0.3)	−0.1 (0.1)
Cz	−1.8 (0.2)	0.4 (0.2)	−1.3 (0.2)	−1.3 (0.2)	1.8 (0.4)	−2.6 (0.1)
Pz	−1.3 (0.2)	1.2 (0.2)	−1.3 (0.2)	−1.1 (0.2)	1.5 (0.4)	−0.9 (0.1)
F3	−1.6 (0.2)	0.7 (0.2)	−1.2 (0.2)	−0.3 (0.2)	1.8 (0.3)	−2.3 (0.1)
F4	−1.7 (0.2)	0.0 (0.2)	−1.1 (0.2)	−0.6 (0.2)	1.7 (0.3)	−2.0 (0.1)
**Monkey Y**						
	**S_1_ (n = 9865)**	**S_2_ (n = 9791)**	**S_3_ (n = 9862)**	**S_4_ (n = 9833)**	**D (n = 4272)**	**D_control_ (n = 5454)**
Fz	−3.1 (0.2)	−1.8 (0.2)	−0.8 (0.2)	−0.4 (0.2)	−3.9 (0.2)	−0.3 (0.2)
Cz	−5.3 (0.2)	−2.4 (0.2)	−1.8 (0.2)	−1.0 (0.2)	−5.5 (0.3)	−0.8 (0.3)
Pz	−3.4 (0.2)	−2.0 (0.3)	−2.8 (0.3)	−1.8 (0.2)	−2.9 (0.4)	−0.4 (0.4)
F3	−2.6 (0.2)	−1.6 (0.2)	−0.8 (0.2)	−0.9 (0.2)	−2.9 (0.3)	−0.4 (0.2)
F4	−2.4 (0.2)	−1.3 (0.2)	−0.9 (0.2)	−0.3 (0.2)	−2.9 (0.3)	−0.3 (0.2)

*Note*. Mean amplitudes (µV) are indicated with *SE* values in parentheses. S_1–4_: values for standard stimuli; D: values for deviant stimuli; D_control:_ values for deviant- control stimuli; n: number of epochs. The time windows adopted are 105–155 ms for monkey A and 73–123 ms for monkey Y (See Methods).

**Table 4:** Mean amplitudes of standard- (S_1–4_), deviant- (D), and ‘deviant-control’-waves (D_control_) in the late window (just after the first sound) for each stimulus type and scalp position (Experiment 3).

Monkey A						
	S_1_ (n = 5458)	S_2_ (n = 5518)	S_3_ (n = 5515)	S_4_ (n = 5421)	D (n = 2404)	D_control_ (n = 12959)
Fz	−0.6 (0.2)	−2.8 (0.2)	−0.9 (0.2)	−0.7 (0.2)	−0.8 (0.4)	−5.0 (0.1)
Cz	−0.8 (0.2)	−2.2 (0.2)	−0.1(0.2)	−0.7 (0.2)	−1.0 (0.4)	−3.1 (0.1)
Pz	−0.2 (0.2)	−1.6 (0.2)	−0.6 (0.2)	−0.3 (0.2)	−0.4 (0.4)	−1.0 (0.1)
F3	−0.6 (0.2)	−2.4 (0.2)	−0.8 (0.2)	−1.1 (0.2)	−0.9 (0.3)	−4.0 (0.1)
F4	−0.7 (0.2)	−1.4 (0.2)	−0.4 (0.2)	−0.4 (0.2)	−0.8 (0.3)	−2.8 (0.1)
**Monkey Y**						
	**S_1_ (n = 9865)**	**S_2_ (n = 9791)**	**S_3_ (n = 9862)**	**S_4_ (n = 9833)**	**D (n = 4272)**	**D_control_ (n = 5454)**
Fz	−1.9 (0.2)	−3.9 (0.2)	−3.9 (0.2)	−1.9 (0.2)	−1.9 (0.2)	−11.4 (0.3)
Cz	−2.4 (0.2)	−5.9 (0.2)	−5.8 (0.2)	−3.5 (0.2)	−2.8 (0.4)	−13.4 (0.4)
Pz	−1.5 (0.3)	−2.7 (0.3)	−2.8 (0.3)	−1.6 (0.3)	−2.3 (0.4)	−2.3 (0.4)
F3	−1.7 (0.2)	−2.5 (0.2)	−3.4 (0.2)	−1.4 (0.2)	−1.3 (0.3)	−6.9 (0.2)
F4	−1.2 (0.2)	−2.6 (0.2)	−2.7 (0.2)	−1.1 (0.2)	−1.6 (0.3)	−5.1 (0.2)

*Note*. Mean amplitudes (µV) are indicated with *SE* values in parentheses. S_1–4_: values for standard stimuli; D: values for deviant stimuli; D_control:_ values for deviant- control stimuli; n: number of epochs. The time windows adopted are 214–264 ms for monkey A and 220–270 ms for monkey Y (See Methods).

**Figure 3:**
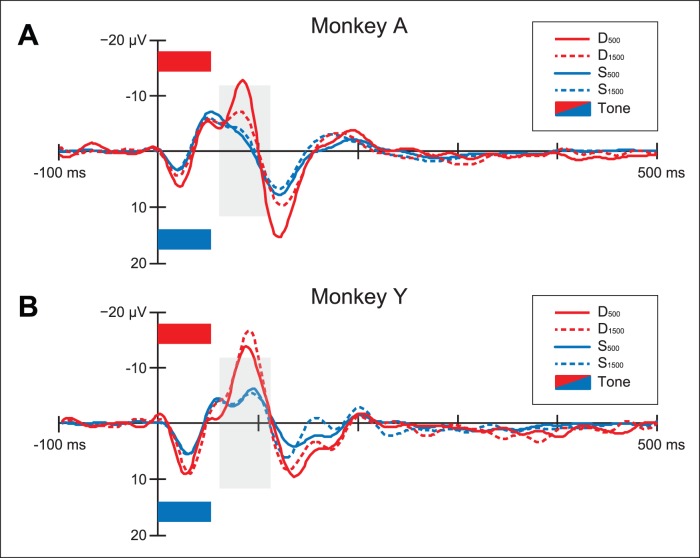
Event-related potentials at Cz for Experiment 1. Zero-aligned ERP responses for standard (S_500_, S_1500_) and deviant (D_500_, D_1500_) tones for monkey A and monkey Y. Stimulus positions are marked with rectangles; The gray-shaded areas indicate the time windows used in the statistical analysis (See Table 1 for details on the time ranges used).

**Figure 4:**
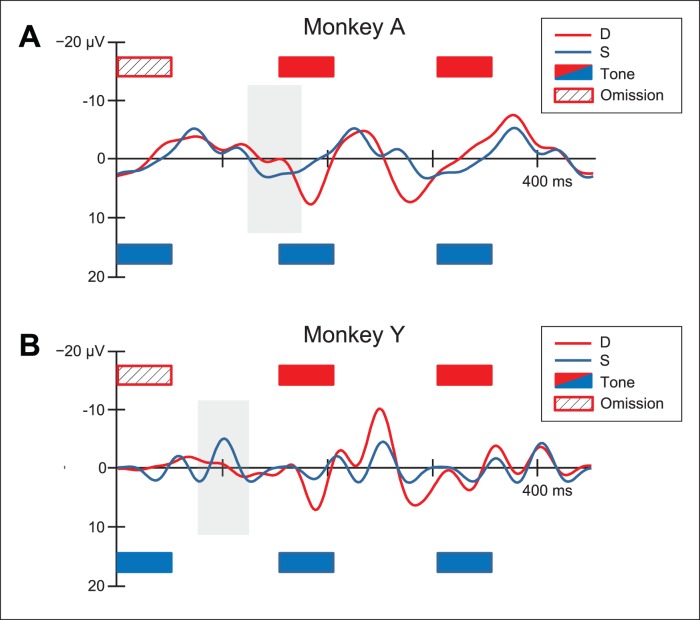
Event-related potentials at Cz for Experiment 2. Zero-aligned ERP responses for standard (tone) and deviant (omission) for monkey A and monkey Y. Stimulus positions are marked with rectangles; The gray-shaded areas indicate the time windows used in the statistical analysis (See Table 2 for details on the time ranges used).

**Figure 5:**
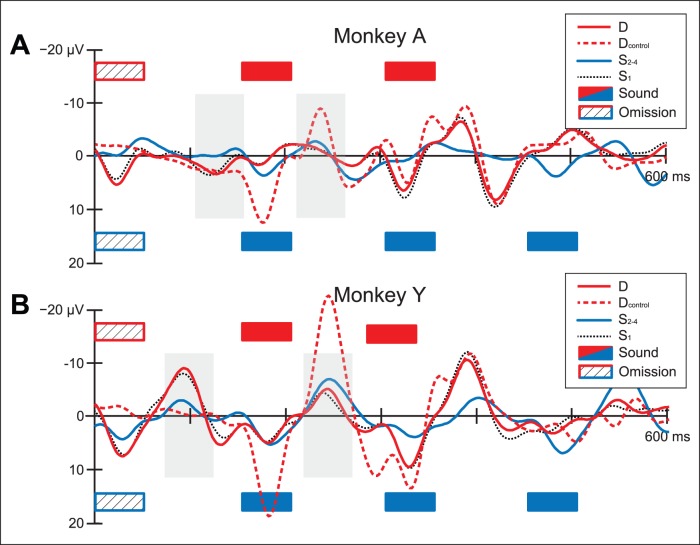
Event-related potentials at Cz for Experiment 3. Omission-aligned ERP responses for the standard (S_2_–S_4_; solid blue line), deviant (D; solid red line), and deviant-control (D_control_; dashed red line). The standard without omission (S_1_; dotted black line) is shown zero-aligned with both deviants (D and D_control_) for comparison. The gray-shaded areas indicate the time windows used in the statistical analysis (See Tables 3 and 4 for details on the time ranges used).

Statistical analysis was performed on the mean amplitudes in a 50 ms wide time window centered on the absolute maximum peak of difference waveforms (i.e. the difference between the standard and deviant wave). The resulting windows are stated underneath the Tables 1 to 4 and marked with gray-shaded rectangles in Figures 3, 4, 5. In all three experiments channel Cz was used for the latency measurements.

The resulting values were fed into an analysis of variance (ANOVA), where Electrode sites were treated as a within subject variable and all other variables as grouping variables. For Experiment 1 factors Stimulus (500 Hz vs. 1500 Hz) × Type (Deviant vs. Standard) × Electrode (Fz vs. Cz vs. Pz vs. F3 vs. F4) were used, for Experiment 2 Type (Omission vs. Sound) × Electrode (Fz vs. Cz vs. Pz vs. F3 vs. F4), and for Experiment 3 Type (Deviant vs. Deviant control vs. S_1–4_) × Electrode (Fz vs. Cz vs. Pz vs. F3 vs. F4). Greenhouse-Geisser correction was used where necessary (corrected p, df and epsilon values reported).

## Results

### Pitch Deviants Evoke an MMN-like Response

In Experiment 1 we presented two rhesus monkeys with a sequence of sounds using a two-tone oddball paradigm (see Methods) to see whether an MMN-like response can be elicited.

Figure 3 shows that the electrical brain responses elicited by the standard and deviant stimulus are different for both monkeys, with a morphology comparable to a human MMN, though with a shorter latency (peaks around 90 ms, instead of 150 ms) and slightly larger amplitude as compared to humans (around 10 µV, instead of 5 µV) [23]. These differences in latency and amplitude can be attributed to the anatomical differences between human and monkey brains (e.g., skull size, thickness, and the distribution of musculature [24]).

For monkey A the ANOVA with factors Stimulus (500 Hz vs. 1500 Hz) × Type (Deviant vs. Standard) × Electrode (Fz vs. Cz vs. Pz vs. F3 vs. F4) revealed significant main effects in Type (*F* (1, 47395) = 104.555, *P*<0.001, *η^2^* = 0.002), Stimulus (*F* (1, 47395) = 12.045, *P*<0.001, *η^2^*<0.001) and Electrode (*F* (3.202, 151750.6) = 151.684, *P*<0.0001, *η^2^* = 0.002, ε = 0.800) as well al a Stimulus × Type interaction (*F* (1, 47395) = 31.476, *P*<0.001, *η^2^* = 0.003). All interactions involving the Electrode factor were significant, namely Electrode × Stimulus (*F* (3.202, 151750.6) = 2.723, *P*<0.05, *η^2^* = 0.002, ε = 0.800), Electrode × Type (*F* (3.202, 151750.6) = 51.294, *P*<0.0001, *η^2^*<0.001, ε = 0.800) and Electrode × Stimulus × Type (*F* (3.202, 151750.6) = 3.113, *P*<0.01, *η^2^*<0.05, ε = 0.800). Tukey unequal-N HSD post-hoc tests revealed no significant difference between F3 and F4 and and Type having no effect on Pz. Additionally the effect of Type was only marginally significant (*df* = 47395, *P* = 0.066) on 1500 Hz stimuli.

For monkey Y the mean negative amplitude for deviant stimuli was significantly greater than that for standard stimuli. An ANOVA with the same factors revealed significant main effects in Type (*F* (1, 78644) = 206.474, *P*<0.001, *η^2^* = 0.003) and Electrode (*F* (2.336, 163892.2) = 181.928, *P*<0.0001, *η^2^* = 0.002, ε = 0.584). All interactions involving the Electrode factor were significant, namely Electrode × Stimulus (*F* (2.336, 163892.2) = 3.543, *P*<0.05, *η^2^* = 0.002, ε = 0.584), Electrode × Type (*F* (2.336, 163892.2) = 35.920, *P*<0.0001, *η^2^*<0.001, ε = 0.584) and Electrode × Stimulus × Type (*F* (2.336, 163892.2) = 6.034, *P*<0.0001, *η^2^*<0.001, ε = 0.584). Tukey unequal-N HSD post-hoc tests revealed no difference between F3 and F4 and Type having no effect on Pz.

An MMN-like response was found for the deviant responses as compared to physically identical standards in a time-window centered on the absolute maximum of the difference waves (D_500_–S_500_, D_1500_–S_1500_; See Table 1 and gray-shaded windows in Figure 3).

The results show that physically identical deviant and standard stimuli elicited different responses. The average amplitude of the responses for both monkeys tended to be large in the frontal and central areas, similar to a human MMN [23]. Table 1 shows the mean amplitudes for monkey A and monkey Y, for each condition, stimulus type and electrode position. There was no indication of hemispheric differences.

These results are in line with another study showing an MMN-like response in a single chimpanzee (*Pan troglodyte*) [16] using the same two-tone odd-ball paradigm with scalp-recorded EEG. Together with the current experiment these studies provide evidence that ERP and MMN can be measured in both monkeys and apes.

### Omissions Evoke an MMN-like Response

To study whether an MMN can be elicited in response to omissions as well, the same rhesus monkeys were presented with a tone sequence in which tones were omitted (i.e. replaced by silence, see Methods).

Figure 4 shows the electrical brain responses elicited by the standard (S) and the deviant (D; an omission). (Note that Figure 4 shows a time window with three repetitions of the standard tone, marked by rectangles at either side of the time line.) This allows for a comparison of the responses to the first and second tone after the omission. To test the effects of the omission we concentrate on the time range closest to the occurrence of the omission (see Methods; Table 2). In both monkeys the standard stimuli elicit a steady-state response with increased amplitude, phase-aligned to the stimuli. The amplitude of the response for the first tone after the omission (see Figure 4), most notably in monkey Y, neural activity increased after the short period of silence, but returns near to previous levels by the second tone. This could also be interpreted as a response marking the beginning of a rhythmic group [25].

Mean amplitudes of responses elicited by standard and deviant stimuli were measured within a time window centered on the absolute maximum of the D minus S difference waves (see Table 2 and gray-shaded windows in Figure 4).

For monkey A an ANOVA with factors Type (Omission vs. Tone) × Electrode (Fz vs. Cz vs. Pz vs. F3 vs. F4) revealed significant main effects in Type (*F* (1, 15708) = 32.906, *P*<0.0001, *η^2^* = 0.002) and Electrode (*F* (2.894, 45465.48) = 32.049, *P<*0.001, *η^2^* = 0.002, ε = 0.724). Tukey unequal-N HSD post-hoc tests revealed no significant difference between F3 and F4.

For monkey Y an ANOVA with the same factors revealed significant main effects in Type (*F* (1, 27307) = 10.648, *P*<0.005, *η^2^*<0.001) and Electrode (*F* (2.255, 61581.99) = 7,477, *P<*0.001, *η^2^*<0.001, ε = 0.564). Tukey unequal-N HSD post-hoc tests revealed no significant difference between F3 and F4.

Again for both monkeys the average amplitude tended to be large in the frontal and central areas, without any laterality effects.

The ERP responses to the omission (red lines in Figure 4) have a morphology comparable to human MMN (i.e. negative in early latencies). However, the polarity of the responses, probably due to inter-individual differences, were different in the two monkeys. Nevertheless, there is a small, but significant amplitude difference between the standard tone and the omission in a time range comparable to human MMN [21], [26] suggesting that the omission was indeed detected.

### Rhesus Monkeys do not Detect ‘Loud Rests’, but are Sensitive to Rhythmic Grouping

In Experiment 3 we presented the same two rhesus monkeys with complex stimuli consisting of sound sequences based on a typical rock drum accompaniment pattern (see Figure 1).

The standard stimuli are four randomly presented and strictly metrical sound patterns (S_1_–S_4_), with a deviant pattern (D) presented which the ‘downbeat’ omitted. Humans adults perceive the D pattern within the context of standards as if the rhythm was broken, stumbled, or became strongly syncopated for a moment [20]. We refer to the omission at the start of D as a ‘loud rest’ and the omissions in S_2_–S_4_ as ‘silent rests’; Music theory suggests the former to sound ‘syncopated’ (a violation of a metric expectation) and the latter not [3].

A sequence repeating the D pattern 100% of the time was also presented (‘deviant-control’ or D_control_) to allow controlling for acoustic effects on the ERP.

On the basis of the *dissociation hypothesis*, and the observation that monkeys apparently can not synchronize to a beat [7] but are sensitive to auditory timing [12], one might expect that monkeys are sensitive to rhythmic structure (interval-based timing) but not to metric structure (beat-based timing). This hypothesis predicts that omissions that play a role in rhythmic grouping [27] can be detected, as they mark the structure of a rhythmic pattern (as is the case in D_control_), consequently not eliciting an MMN as they are part of the regularity. In contrast, the omissions that do not affect the rhythmic grouping will not be detected as part of a regularity, since they occur irregularly (as is the case in S_2_–S_4_ and D) and hence may elicit an MMN.

In humans these differences in salience appear to be related to the coding of an internal representation of the rhythmic structure of a sound pattern [27], with the first sound *after* a relatively long inter-onset interval determining the rhythmic group structure [25]. If this is the case we expect the first sound of a repeated rhythmic pattern (D_control_) – but not a randomly inserted pattern (D) – to elicit a response marking the beginning of a rhythmic group [25].

An alternative hypothesis is based on the observations made in human adults and newborns using the same stimuli and experimental paradigm [17], [18], [19], [20]. This hypothesis predicts that primates are not only able to sense rhythmic grouping, but are also able to detect the regular beat that is induced by a varying rhythmic stimulus. The perception of a ‘loud rest’ – a violation of a temporal expectation reflected by an MMN-like signal– can serve as evidence for the presence of a strong metric expectation [3]. This hypothesis predicts an large and early MMN for the omission in the deviant (D, containing a ‘loud rest’), but no or considerably smaller MMN for the omissions in the standard (S_2_–S_4,_ containing ‘silent rests’). And since the omission in the deviant-control (D_control_) is expected – the pattern is presented repeatedly –, there as well no MMN is predicted. If these three aspects are observed (as they were found in human adults and newborns [20]), they suggest that a regular beat is extracted from the auditory stimulus. This could be interpreted as evidence against the vocal learning hypothesis.

Figure 5 shows that the electrical brain responses elicited by omissions in the standard (S_2_–S_4_) and deviant-control (D*_control_*) are relatively flat, and different from the deviant (D), with the latter eliciting a more pronounced negative peak, most notably in monkey Y. This suggest a similar result as was found human adults and newborns. However, the ERP response to S_1_ (dotted black line in Figure 5) is not different from that in response to D (solid red line in Figure 5), while D contains an omission and S_1_ does not. This seriously weakens the interpretation that the monkeys are able to extract the beat from the stimulus.

Mean amplitudes of responses elicited by standard and deviant stimuli were measured within a time window centered on the absolute maximum of the D minus S_2–4_ difference waves (see Table 3 and the early gray-shaded windows in Figure 5).

For monkey A an ANOVA with factors Type (S_1_ vs. S_2_ vs. S_3_ vs. S_4_ vs. D_control_ vs. D) × Electrode (Fz vs. Cz vs. Pz vs. F3 vs. F4) in the early window (105–155 ms) showed significant main effects in Type (*F* (5, 37269) = 89.318, *P*<0.0001, *η^2^* = 0.012) and Electrode (*F* (3.006, 112063.6) = 11.221, *P*<0.0001, η*^2^*<0.001, ε = 0.752), as well as a significant Electrode × Type (*F* (15.034, 112063.6) = 7.475, *P*<0.0001, *η^2^* = 0.001, ε = 0.752) interaction. Tukey unequal-N HSD post-hoc tests were performed. All channels differed from each other (*df* = 149076, *P*<0.05) except for Cz, F3 and F4 not differing from each other. All Types differed from each other (*df* = 49071, *P*<0.01), except D, D_control_ and S_1_ from each other and S_3_ from S_4_.

For monkey Y an ANOVA with factors Type (S_1_ vs. S_2_ vs. S_3_ vs. S_4_ vs. D_control_ vs. D) × Electrode (Fz vs. Cz vs. Pz vs. F3 vs. F4) in the early window (73–123 ms) showed significant main effects in Type (*F* (5, 49071) = 74.323, *P*<0.0001, *η^2^* = 0.008) and Electrode (*F* (2.412, 118344.7) = 48.423, *P*<0.0001, η*^2^* = 0.001, ε = 0.603), as well as a significant Electrode × Type (*F* (12.059, 118344.7) = 9.479, *P*<0.0001, *η^2^* = 0.001, ε = 0.603) interaction. Tukey unequal-N HSD post-hoc tests were performed. All channels differed from each other (*df* = 196284, *P*<0.05) except for F3 and F4 and Fz not differing from F3. All Types differed from each other (*df* = 49071, *P*<0.001), except D from S_1_; S_2_ from S_3_ and S_4_ from D_control_ also the difference between S_3_ and S_4_ was less significant (*P*<0.05) than other differences.

So in short, while there is a difference between D (containing a ‘loud rest’) and S_2_–S_4_ (containing ‘silent rests’) and as such evidence in support of beat perception, there is no difference between D and S_1_: a pattern with and without an omission. This makes the interpretation that the monkeys are detecting the beat (by distinguishing ‘loud rests’ from ‘silent rests’) less likely and leads to the alternative hypothesis that the monkeys are solely detecting rhythmic groups [21]–[22]: the first note of a rhythmic group (separated by an omission) eliciting an MMN-like response in D_control_ (but not in D).

Mean amplitudes were measured in a late time window just after the first tone (after 200 ms), centered on the absolute maximum of the D minus D_control_ difference waves (see Table 4 and the late gray-shaded windows in Figure 5).

For monkey A the ANOVA with the same factors on the late window (214–264 ms) showed significant main effects in Type (*F* (5, 49071) = 71.134, *P*<0.0001, *η^2^* = 0.009) and Electrode (*F* (2.975, 110879.9) = 35.850, *P*<0.0001, η*^2^*<0.001, ε = 0.744), as well as a significant Electrode × Type (*F* (14.876, 110879.9) = 19.880, *P*<0.0001, *η^2^* = 0.003, ε = 0.744) interaction. Tukey unequal-N HSD post-hoc tests were performed showing that D was significantly different from D_control_ (*df* = 37269, *P*<0.001) while not differing from S_1_. All channels differed from each other (*df* = 149076, *P*<0.001) except for Cz and F4.

For monkey Y the ANOVA with the same factors on the late window (220–270 ms) showed significant main effects in Type (*F* (5, 49071) = 195.816, *P*<0.0001, *η^2^* = 0.020) and Electrode (*F* (2.412, 118344.7) = 283.270, *P*<0.0001, η*^2^* = 0.006, ε = 0.604), as well as a significant Electrode × Type (*F* (12.059, 118344.7) = 47.789, *P*<0.0001, *η^2^* = 0.005, ε = 0.604) interaction. Tukey unequal-N HSD post-hoc tests were performed showing that D was significantly different from D_control_ (*df* = 49071, *P*<0.001) while not differing from S_1_. All channels differed from each other (*df* = 196284, *P*<0.001) except for Pz and F4.

These results suggests that the monkeys are actually sensing surface-level rhythmic grouping (i.e. detecting the start of a repeating rhythmic group) instead of the induced beat (i.e. detecting a regular pulse in a varying rhythmic pattern). As such, we have to conclude that rhesus monkeys, contrary to what has been shown for human adults and newborns, show no sign of representing the beat in music, but apparently do represent rhythmic groups.

## Discussion and Conclusion

Electrophysiological measures such as event-related brain potentials (ERP) are a useful tool in the study of beat induction the metrical encoding of rhythm, especially in examining its predictive nature [3]. An informative component of ERP is the mismatch negativity (MMN): a negative deflection in the brain signal that occurs if something unexpected happens while listening (even during passive listening) [23]. This MMN is generally thought to reflect an error signal that is elicited when incoming sensory information does not match the expectations created by previous information. Also abstract information (i.e. one auditory feature predicting another) and omissions [21], [26] can cause an MMN, resulting in an interpretation of the MMN as reflecting the detection of regularity-violations as part of a predictive process, rather than just sample matching to sensory memory [28].

In the current study we demonstrate for the first time that an MMN-like ERP component can be measured in rhesus monkeys *(Macaca mulatta)*, both for pitch deviants (Experiment 1) and omissions (Experiment 2). Together these results provide support for the idea that ERP and MMN can be used as an index of the detection of regularity-violations in an auditory signal in rhesus monkeys.

In addition, we showed that rhesus monkeys are not able to detect the regularity induced by a varying rhythm, while being sensitive to the rhythmic grouping structure. These findings are in support of the hypothesis that *beat induction* (the cognitive mechanism that supports the perception of a regular pulse from a varying rhythm) is species-specific, and it is likely restricted to vocal learners such as a selected group of bird species [4], while absent in nonhuman primates such as rhesus monkeys [7]. This is evidence in support of the *vocal learning hypothesis*.

Furthermore, the results are in line with the auditory timing *dissociation hypothesis*, suggesting rhythm perception to be distinct from beat perception [10], [11], [12]. However, the current paradigm, with just a few electrodes measuring EEG, does not allow us to say anything about the brain networks that might be involved. For this fMRI and other brain imaging techniques with a high spatial resolution are needed [29].

And finally, the current study suggests, together with the few existing studies on auditory [16] and visual [30] processing in monkeys, EEG to be a worthwhile, non-invasive alternative in the study of cognitive and neural processing in primates.
